# Plant Life in Extreme Environments: How Do You Improve Drought Tolerance?

**DOI:** 10.3389/fpls.2018.00543

**Published:** 2018-05-15

**Authors:** Ulrike Bechtold

**Affiliations:** School of Biological Sciences, University of Essex, Colchester, United Kingdom

**Keywords:** extremophiles, Arabidopsis, drought survival, drought tolerance, drought avoidance

## Abstract

Systems studies of drought stress in resurrection plants and other xerophytes are rapidly identifying a large number of genes, proteins and metabolites that respond to severe drought stress or desiccation. This has provided insight into drought resistance mechanisms, which allow xerophytes to persist under such extreme environmental conditions. Some of the mechanisms that ensure cellular protection during severe dehydration appear to be unique to desert species, while many other stress signaling pathways are in common with well-studied model and crop species. However, despite the identification of many desiccation inducible genes, there are few “gene-to-field” examples that have led to improved drought tolerance and yield stability derived from resurrection plants, and only few examples have emerged from model species. This has led to many critical reviews on the merit of the experimental approaches and the type of plants used to study drought resistance mechanisms. This article discusses the long-standing arguments between the ecophysiology and molecular biology communities, on how to “drought-proof” future crop varieties. It concludes that a more positive and inclusive dialogue between the different disciplines is needed, to allow us to move forward in a much more constructive way.

## Introduction

Four hundred and fifty million years of land plant evolution has generated biological complexity, which has allowed plants to adapt to terrestrial environments, ranging from extreme cold environments in the Arctic and Antarctica, high salinity environments to extreme temperature changes and drought conditions in desert environments (von Willert et al., 1990; Alberdi et al., 2002; Amtmann et al., 2005). Plants that inhabit these environments are collectively called extremophiles, which harbor a range of different mechanisms that allow them to withstand these extreme environments.

During the green revolution of late twentieth century, breeding dramatically modified plant architecture, and yield improvements were made by selecting for characteristics such as rapid growth and a reduction of vegetative biomass in favor of fruit and seed production (Pingali, 2012). However, artificial selection for yield inadvertently reduced diversity, resulting in the loss of abiotic stress tolerance, with crop species likely to be more sensitive to abiotic stress compared to their wild ancestors (Mayrose et al., 2011; Koziol et al., 2012). Abiotic stresses dramatically reduce crop yields posing a threat to food security (Boyer, 1982; Cramer et al., 2011), and with the worldwide population growth expected to reach 9.7 billion people by 2050 (United Nations Department of Economic Social Affairs, 2017), the demand for global crop production is expected to double by 2050 (Tilman et al., 2011). This problem is likely to be exacerbated in the future by climate change (Mittler and Blumwald, 2010; Lesk et al., 2016). Furthermore future expansion of agricultural area is likely to occur in drylands and deserts (Millennium Ecosystem Assessment, 2005; Millenium Ecosystem Assessment, 2010), and new solutions to meet the world's future food security by improving crop yields is vital to not only prevent losses where crops are currently grown but also to cultivate them on more marginal land (Foley et al., 2011).

Interestingly, just as the plant stress community is beginning to tackle the molecular mechanisms of combined stress tolerances in models, crops and extremophiles, crop ecophysiologists are reassessing how misconceptions of stress resistance mechanisms may be avoided, advocating the need for clear physiological frameworks to meaningfully integrate the wealth of genetic response data. This article will focus on the efforts being made to understand dehydration resistance mechanisms in extremophile and model plants, and discuss the prospects of unlocking the genetic codes and mechanisms of extremophiles in the battle for stress tolerant crops.

## Why are resurrection plants so superb at surviving drought stress?

Until recently, our knowledge of the molecular mechanisms of stress tolerance in extremophiles was relatively limited, but with the onset of next generation sequencing technologies, the number genome and transcriptome datasets of extremophiles has steadily increased (Oh et al., 2012; Dinakar and Bartels, 2013). In this context, halophytes have traditionally attracted more attention at the molecular level, partly because they include highly salt tolerant close relatives of *Arabidopsis thaliana*, such as *Thellungiella parvula* and *Eutrema salsugineum* (Dassanayake et al., 2011; Wu et al., 2012), allowing for direct comparisons of stress tolerance mechanisms such as salt, cold, heat, drought, and freezing tolerances already widely studied in Arabidopsis (Lee et al., 2012; Koch and German, 2013). *E. salsugineum* also harbors tolerances to low soil nitrogen (Kant et al., 2008), high boron levels (Lamdan et al., 2012), low phosphate levels (Velasco et al., 2016) and heat stress (Higashi et al., 2013), which is an exciting prospect for translating multiple stress tolerance traits to other plant species, including agronomically relevant *Brassicaceae*.

The interest in vegetative desiccation tolerance is illustrated by the large the number of publications relating to their ecology, physiology and molecular mechanism over the last 20 years, with 700 publications containing a reference to desiccation tolerance in plants, of which 257 were linked to resurrection plants (https://www.ncbi.nlm.nih.gov/pubmed/, search terms: desiccation tolerance, resurrection plants, last searched 3/2/18). With more than 130 known varieties, resurrection plants are mostly found in arid and semi-arid environments, (Gaff, 1971, 1977), and are probably the best studied of the xerophytes. Common to all resurrection plants is a vegetative desiccation tolerance (Oliver et al., 2000), and a small number of different species have been extensively studied with the aim to identify the underlying molecular mechanisms (Farrant, 2000; Bartels and Salamini, 2001; Cooper, 2002; Bartels, 2005; Farrant et al., 2015). Resurrection plants rapidly respond to water deficiency by switching into a “*stress mode*” that leads to a complete inhibition of photosynthesis and an overproduction of reactive oxygen species (ROS) in the chloroplasts due to excess light energy. ROS subsequently oxidize proteins and lipids, damage DNA and RNA, and ultimately lead to programmed cell death (Farrant et al., 2003).

Transcriptome analysis of *Craterostigma plantagineum* and *Haberlea rhodopensis* (Rodriguez et al., 2010; Gechev et al., 2013; Giarola et al., 2017) under early dehydration, desiccation and subsequent rehydration revealed common genetic pathways among desiccation-tolerant species. Resurrection plants undergo different stages during desiccation, which are accompanied by distinct physiological, metabolic, and molecular changes extensively reviewed by Farrant et al. (2015). Essentially, during the early dehydration stage, photosynthesis is shut down, ABA dependent responses to water stress become prominent, there is increased activity of antioxidants and a redirection of metabolism to the increased formation of sucrose and oligosaccharides as osmo-protectants (Rodriguez et al., 2010; Farrant et al., 2015). The late stages of drying are indicated by increased expression of proteins involved in signal transduction, altering sugar metabolism and genes encoding classical stress-associated proteins such as early light-inducible (ELIPs), Heat Shock Proteins (HSPs) and Late Embryogenesis Abundant (LEAs) proteins (Gechev et al., 2013; Farrant et al., 2015; Costa et al., 2017). This reprogramming of metabolism is driven by the induction of known stress responsive transcription factors, such as NACs, NF-Ys, HSFs, and WRKYs (Gechev et al., 2013; Farrant et al., 2015; Costa et al., 2017). It appears that resurrection plants are generally in a constantly “primed state,” with high basal levels of protective sugars, antioxidants and defense proteins, allowing a rapid and strong response during desiccation (Rodriguez et al., 2010; Gechev et al., 2013; Costa et al., 2016).

## What are the lessons learned from resurrection plants?

Many of the genes identified in the above transcriptome studies have also been identified in drought responses of Arabidopsis and other plant models (Tripathi et al., 2014; Bechtold et al., 2016), suggesting that common signaling pathways are in operation. The advantages of Arabidopsis as a model for plant molecular biology and the role it played and still plays in investigating abiotic stress response pathways is undisputed (Provart et al., 2016). Many of the stress signaling pathways identified are now known to be general responses that appear to be conserved in many higher plant species (Boscaiu et al., 2012; Provart et al., 2016).

However, there are counterarguments which suggest that too much emphasis is being placed on investigating unsuitable experimental models, such as Arabidopsis (Boscaiu et al., 2012). For example, Arabidopsis and many crop plants die at leaf water potentials of around −3 MPa (van der Weele et al., 2000; Fitter and Hay, 2002). Consequently, Arabidopsis may not be appropriate to identify dehydration tolerance pathways. Yet most of the biochemical and molecular studies on plant responses to abiotic stress have been carried out using Arabidopsis (Bressan et al., 2009), which has resulted in the identification and isolation of stress-tolerance genes, some of which have been used in modulating crop stress tolerance with varying success (reviewed by Mittler and Blumwald, 2010; Varshney et al., 2011). Especially stress responsive transcription factors (TFs) such as the AP2/EREBP family DREBs, MYB, WRKY, NAC, bHLH, and bZIPs have attracted attention due to their important roles in plant stress responses and improved stress tolerance phenotypes when overexpressed in Arabidopsis and crop plants alike (reviewed by Wang et al., 2016).

From many of these transgenic studies it is evident that TFs have conserved functions across species boundaries including resurrection plants (see above), and many of the Arabidopsis TFs have been shown to confer stress tolerances in unrelated crop species and vice versa (Jiang et al., 2011; Wang et al., 2016). However, the bottom line is that currently few abiotic stress tolerant, high yielding crops are grown in our fields utilizing the genes identified from the many biochemical and molecular analyses carried out on Arabidopsis and/or model crops reviewed by (Mickelbart et al., 2015; Ricroch and Hénard-Damave, 2016).

This has raised several questions with regard to the experimental conditions applied in gene discovery studies, the appropriateness of the stress phenotypes being assessed (Blum and Tuberosa, 2018), or whether common stress signaling pathways are indeed the best targets (Boscaiu et al., 2012). While vegetative desiccation tolerance in resurrection plants is clearly at the extreme end of the stress survival spectrum, it is argued that extremophile species may act as a source of novel genes for the genetic improvement of stress tolerance in crops (Boscaiu et al., 2012). Yet many of the TF families identified in Arabidopsis have also been identified in resurrection plants in response to drought stress (see above; Gechev et al., 2013; Farrant et al., 2015; Costa et al., 2017). It is now generally accepted that many of these common signaling pathways are functional in xerophytes adapted to arid or semi-arid conditions (Farrant et al., 2015; Costa et al., 2016, 2017). Therefore, the argument regarding the suitability of stress sensitive model species vs. xerophytes/resurrection plants in the pursuit to study drought responses is unresolved, and the question remains how studies on resurrection plants are going to lead us to novel genes and stress signaling pathways, when so far, many transcriptome studies have mostly delivered on general stress pathways?

Interestingly, transcriptome studies of resurrection plants not only found a high proportion of unknown transcripts (33% *C. plantagineum* and ~40% *H. rhodopensis;* Rodriguez et al., 2010; Gechev et al., 2013), but in the case of the *C. plantagineum* transcriptome, also identified many taxonomically restricted genes (TRGs) and non-protein coding RNAs (ncRNAs) (Giarola et al., 2014; Giarola and Bartels, 2015). TRGs are known to code for new traits required for the adaptation of organisms to particular environmental conditions (Johnson and Tsutsui, 2011), and it has been suggested that these may harbor the potential for novel gene discovery linked to desiccation tolerance.

Early attempts to utilize TRGs such as desiccation induced proteins from *C. plantagineum* to improve drought tolerance in tobacco failed (Iturriaga et al., 1992), while recent examples successfully used tonoplast cation/H+ antiporter and H+ pyrophophatases genes from the xerophyte *Zygophyllum xanthoxylum* to enhance stress tolerance in alfalfa and *Lotus corniculatus* (Bao et al., 2014, 2016). These studies suggested that improved “systems wide approaches” of large datasets, together with comparative genomics that aim to identify whole network-based homologies between species could be more successful in discovering novel genes/pathways that underlie differences and similarities across species (Farrant et al., 2015).

## The continuous argument—are we studying the right system?

There also appears to be a lack of synergy between different disciplines with physiology, ecophysiology on the one hand, and genetics and molecular biology on the other. Perhaps the problem is not only the type of plant we study, but also how we study stress tolerance mechanisms in general? Crop physiologists rightly argue that molecular mechanisms of plant survival traits are often studied in isolation to physiological responses, whether this is carried out in model species such as Arabidopsis (Blum, 2005), or in extremophiles such as the resurrection plants (Blum and Tuberosa, 2018). This may be confounded by experimental setups that are not fit for purpose or comparable to plant responses under field conditions, for example pot grown vs. field grown plants (Passioura J. B., 2006; Poorter et al., 2012).

Desiccation tolerance in resurrection plants is a survival trait, and survival traits after any given stress have generally been a popular phenotype for gene discovery and gene function in model species (Woo et al., 2008; Skirycz et al., 2011), and drought resistance is often assessed under quite severe conditions in which plant survival is scored after a prolonged period of soil drying. Yet, even with what superficially appears to be simple phenotype (i.e., survival); the mechanisms ensuring drought survival are often no being fully assessed (Blum and Tuberosa, 2018). Plant survival after a period of drought stress, can either be due to dehydration avoidance or dehydration tolerance (Levitt, 1980). Avoidance strategies are observed in plants that maintain high plant water status due to osmotic adjustment during dehydration, while tolerance usually result from a delayed mortality in response to low plant water status (Levitt, 1980). In resurrection plants, the desiccation survival trait is due to delayed mortality and suppression of drought-related senescence pathways (Griffiths et al., 2014). Therefore, when phenotyping for dehydration survival there is a need to distinguish between dehydration avoidance and dehydration tolerance, and how this may affect survival and recovery. Without this distinction, it has been argued that molecular studies performed to identify the genes that underlie this trait might be biased. For example, in gene-expression studies, RNA is sampled after a set period of dehydration, or at a given relative soil water content, and often the assumption is made that all genotypes are therefore subjected to the same level of stress (Des Marais et al., 2012). Without additional information regarding the physiological- or plant water status this could result in artifacts, where differences in plant water status on the day of sampling are not adequately taken into account. Consequently, an in depth understanding of the physiological basis of the phenotype is essential to avoid misinterpretations (Zhang et al., 2014; Bechtold et al., 2016). While there have clearly been some deficiencies with molecular biology approaches in many molecular/genomic centric studies used to study drought stress, recent critics of the topic fail to mention the progress made by molecular biologists to address some of these early short-comings in the area. In recent years, much more effort has been placed on linking plant genomic events to the metabolic status and the rate of plant dehydration especially in model species, such as Arabidopsis, *Medicago truncatula* and rice (Harb et al., 2010; Des Marais et al., 2012; Lasky et al., 2014; Zhang et al., 2014; Bechtold et al., 2016; Wilkins et al., 2016), resurrection plants (Farrant et al., 2015) and other extremophiles (Brinker et al., 2010). Often these changes are recorded along a gradient of relative water content, plant water potential, plant physiological or metabolic changes (Brinker et al., 2010; Farrant and Moore, 2011; Zhang et al., 2014; Farrant et al., 2015; Bechtold et al., 2016). For example, a significant relationship between water potential and the number of differentially expressed genes was observed in a progressive drought time-series experiments, allowing the clear distinction between early and late dehydration responses at the transcriptional as well as at the physiological level (Bechtold et al., 2016). More importantly, the switch between early and late dehydration responses coincided with a breakpoint in the soil dehydration profile, and this breakpoint clearly differs between natural accessions of Arabidopsis (Ferguson et al., 2018), suggesting difference in dehydration strategies and potentially drought resistance strategies.

In crop plants drought survival through delayed mortality is unlikely to be a suitable option due to expected growth and yield penalties, and therefore questions regarding the usefulness of survival traits in achieving meaningful improvements still remain (Passioura J., 2006; Skirycz and Inzé, 2010; Blum, 2011; Skirycz et al., 2011). Resurrection plants, although excellent models to investigate drought tolerance strategies associated with delayed mortality, may therefore not be appropriate to investigate avoidance strategies associated with the maintenance of photosynthesis and growth. Consequently, plant species adapted to arid and semi-arid environments that avoid desiccation may be more useful models to study the phenomena of stress tolerance under extreme environmental conditions. One such species is the C3 desert plant *Rhazya stricta*, which is common in arid zones at elevations of 100–700 m above sea level, and overcomes water restriction through long tap roots to access water in underground river beds (Batanouny and Baeshin, 1983).

## The need to study xerophytes in their native environment

Extensive physiological phenotyping under native growth conditions is essential due to the extreme conditions experienced in their native environments. Furthermore, growth conditions in control environments often do not fully reflect the conditions experienced in desert environments, especially with regards to the prevailing light conditions (Table 1), even though light is one of the main contributing factors of chloroplast damage during desiccation (Farrant et al., 2003). Relatively few studies have attempted transcriptome analysis from plants grown in their native environments (Table 1), and even fewer have underpinned these studies with extensive physiological characterization of these plants in their native habitats (Yates et al., 2014).

**Table 1:** Comparison of growth conditions in transcriptome, proteome, metabolome, and physiological studies of extremophiles.

	**Native habitat conditions**	**Growth cabinet conditions**	**Experiment growth conditions**	**Measurements/treatments**	**References**
***ANASTATICA HIEROCHUNTICA***					
	Negev Desert: temperature range−3.6 to 46.8°C, arid	16 h day; 150 μmol m^−2^ s^−1;^ 22°C, RH not specified	Growth cabinet	Metabolic profiling/salt and heat stress (MS agar medium)	Eshel et al., 2017
***EUTREMA SALSUGINEUM***					
Accession: Yukon	Yukon territory: temperature range 15–24°C, light 1,500 μmol m^−2^ s^−1^, semi-arid	21 h day, 250 μmol m^−2^ s^−1^, 22/10°C, RH not specified	Growth cabinet and native habitat	Transcriptome/control vs. native habitat (soil grown)	Champigny et al., 2013
Accession: Shangdon		21 h day, 250 μmol m^−2^ s^−1^, 22/10°C, RH not specified	Growth cabinet	Transcriptome/of natural variation (soil grown)	Champigny et al., 2013
Accession: Yukon	Yukon territory: temperature range 15–24°C, light 1,500 μmol m^−2^ s^−1^, semi-arid	21 h day, 250 μmol m^−2^ s^−1^, 22/10°C, RH not specified	Growth cabinet and native habitat	Transcriptome and metabolome/ control vs. native habitats (soil grown)	Guevara et al., 2012
***RHAZYA STRICTA***					
	Bahrah (Saudi Arabia): day temperature 36–43°C, light >1,000 μmol m^−2^ s^−1^, arid	Not applicable	Native habitat	Diurnal transcriptome	Yates et al., 2014
	Bahrah (Saudi Arabia): day temperature 36–43°C, light >1,000 μmol m^−2^ s^−1^, arid	Not applicable	Native habitat	Diurnal changes in photosynthesis leaf physiology	Lawson et al., 2014
***EUTREMA PARVULUM***					
	Salt flats in Tuz (Central Anatolia, Turkey)	12 h day, 22/20°C, 60%RH, light not specified	Growth cabinet	Chloroplast physiology, salt stress (soil mixture)	Uzilday et al., 2015
***CRATEROSTIGMA PLANTAGINEUM***					
	South Africa (conditions not specified)	16 h day, 4,000 lux, 23/19°C RH not specified	Growth cabinet	Transcriptome/different dehydration levels (artificial clay)	Rodriguez et al., 2010
	South Africa (conditions not specified)	14 h day, 24/20°C, 60,000 lx, 60% RH	Growth cabinet	Expression profile of GRP1/desiccation (clay)	Giarola et al., 2016
	South Africa (conditions not specified)		Growth cabinet	Expression profile of EDR1 and CRP1/dehydration and rehydration (clay)	Giarola et al., 2014
***SPOROBOLUS STAPFIANUS***					
	Verena, Transvaal, South Africa	16 h day, 28/19°C, light and RH not specified	Growth cabinet	Transcriptome and metabolome/dehydration and rehydration (soil)	Yobi et al., 2017
		16 h day, 28/19°C, light and RH not specified	Growth cabinet	Proteome/dehydration (soil)	Oliver et al., 2011b
**SPOROBOLUS PYRAMIDALIS**					
		16 h day, 28/19°C, light and RH not specified	Growth cabinet	Metabolome/during dehydration (soil) Comparison with *S. stapfianus*	Oliver et al., 2011a
***CYNANCHUM KOMAROVII***					
	Yinchuan City, Ningxia, China	16 h day, 28/16°C, natural light (intensity not specified)	Growth cabinet	Transcriptome/drought stress (soil and vermiculite)	Ma et al., 2015
***HABERLEA RHODOPENSIS***					
	Rhodope Mountains, Bulgaria, light at harvesting side 20 μE m^−2^ s^−1^	16 h day, 21°C, 20 μE m^−2^ s^−1^, RH 65%	Growth cabinet	Transcriptome and metabolomics (soil)	Gechev et al., 2013
***ZYGOPHYLLUM XANTHOXYLUM***					
	Desert areas in China and Mongolia	16 h day, 28/23°C, 800 μmol m^−2^ s^−1^, RH 65–70%	Growth cabinet	Transcriptome/ salt and osmotic stress (sand)	Ma et al., 2016
***POPULUS EUPHRATICA***					
	Shapotou Desert Experiment and Research, Ningxia, China	12 h day, 25°C, 70 μmol m^−2^ s^−1^, RH not specified	Growth cabinet	Transcriptome / salt stress (MS agar medium)	Qiu et al., 2011
***CALOTROPIS PROCERA***					
	Saudi Arabia, field site near Jeddah: day temperature 36–43°C, light >1,000 μmol m^−2^ s^−1^, arid	16 h day, 25–28°C, 8,000lx, RH not specified	Growth cabinet	Transcriptome and metabolome/salt and drought stress (growth medium not specified)	Mutwakil et al., 2017
	Vargas State, Venezuela: light 100–1,500 μmol m^−2^ s^−1^, temperature 25–32°C, RH 65–85%	Not applicable	Native habitat	Photosynthetic physiology	Tezara et al., 2011

*RH, relative humidity*.

For example, physiological and transcriptome analysis of *R. stricta* in its native desert environment revealed that *R. stricta* maintains growth and high photosynthetic rates at leaf temperatures as high as 43°C, light intensities >1,000 μmol m^−1^ s^−1^ and high vapor pressure deficits (VPDs; Lawson et al., 2014), and is one of the few desert C3 shrubs that achieve such high photosynthetic yields at leaf temperatures above 40°C (Mooney et al., 1978; Tezara et al., 2011; Rivas et al., 2017). Gene expression and gene sequence analysis identified two *RUBISCO ACTIVASE* isoforms, that are likely responsible for the maintenance of high Rubisco activities and photosynthetic rates under these extreme conditions (Lawson et al., 2014). Interestingly, in this very hot and arid environment, *R. strica* is able to maintain an adequate water supply in order to maintain a high and constant photosynthetic activity (Lawson et al., 2014). While strictly speaking *R. stricta* may not suffer from drought stress in those circumstances, it nevertheless gives us an insight into extreme thermo- and high light tolerance in arid environments, which are often part of drought stress conditions experienced in the field. Importantly, by combining detailed physiological analysis with genetic investigations, it was possible to identify some potentially different physiological adaptation mechanisms that go beyond the usual TFs and chaperones mentioned above. Interestingly, a detailed study of the diurnal transcriptome analysis of *R. stricta*, identified considerable overlap with *C. plantagineum* and *P. euphratica* such as cysteine proteases and raffinose synthesis genes, highlighting more general protective mechanisms against high temperature (Yates et al., 2014). Furthermore, gene families specific to *R. stricta*, such as photosynthesis and respiration associated genes, were differentially expressed at midday during a diurnal period responding to VPD, temperature and light levels (Yates et al., 2014). These changes coincided with changes in photosynthetic physiology (Lawson et al., 2014; Yates et al., 2014). Importantly, a number of these unique protein families were found to have diverged from their homologs in other species (Yates et al., 2014). Therefore, C3 species from arid/semi-arid environments that do not undergo a dormant state, could provide novel gene targets responsible for maintaining photosynthesis more commonly associated with dehydration avoidance.

## Conclusions

With unprecedented access to increasing genome information and transcriptome datasets from a variety of plants, coupled with tools to analyse and compare these datasets, we are beginning to identify gene families and gene regulatory networks behind complex traits such as dehydration tolerance. It is only by developing a more realistic framework in which to study drought resistance mechanism that we will make progress in understanding how plant productivity can be maximized under water limiting conditions. For this to happen, a more positive and inclusive dialogue between the different disciplines, all primarily concerned with “drought-proofing” future crop varieties, is essential in order to move forward in a much more constructive way.

## Author contributions

The author confirms being the sole contributor of this work and approved it for publication.

### Conflict of interest statement

The author declares that the research was conducted in the absence of any commercial or financial relationships that could be construed as a potential conflict of interest.
